# Association between polymorphisms in pre-miRNA genes and risk of oral squamous cell cancer in a Chinese population

**DOI:** 10.1371/journal.pone.0176044

**Published:** 2017-06-13

**Authors:** Enjiao Zhang, Zhongfei Xu, Weiyi Duan, Shaohui Huang, Li Lu

**Affiliations:** 1 Department of Oral and Maxillofacial Surgery, School of Stomatology, China Medical University, Shenyang, PR China; 2 Department of Oromaxillofacial-Head and Neck Surgery, School of Stomatology, China Medical University, Shenyang, PR China; Duke Cancer Institute, UNITED STATES

## Abstract

**Background:**

MicroRNAs play important roles in the development of human cancers. This case-control study is to evaluate the roles of the polymorphisms in pre-miRNAs on risk of oral cancer in a Chinese population.

**Methods:**

The genotypes of three polymorphisms were determined in 340 patients with oral squamous cell cancer and 340 healthy controls who were frequency matched for age and sex. Odds ratios (ORs) and 95% confidence intervals (95%CIs) were calculated to assess the association. All analyses were performed using the SPSS software. 3.154() 0.001.

**Results:**

For miR-499 rs3746444, individuals carrying homozygous CC genotype had increased risks of oral cancer compared with the homozygous wild TT genotype (adjusted OR was 3.154, 95%CI was 1.555–6.397, P value was 0.001). The C allele of miR-499 rs3746444 was associated with a higher risk of oral cancer with significant odds ratio of 1.453. In the stratified analyses by sex, the associations between miR-499 rs3746444 and miR-146a rs2910164 polymorphisms with the susceptibility of oral squamous cell cancer were significant in males. However, with 1/4 as many subjects there were no significant associations between the three polymorphisms and oral cancer risks in females. The joint effects of miRNA polymorphisms and smoking on the risk of OSCC were analyzed and the results suggested that the association between microRNA genetic variants and OSCC risk was modified by smoking.

**Conclusions:**

These findings suggest that miR-499 rs3746444 and miR-146a rs2910164 polymorphisms may contribute to genetic susceptibility to oral squamous cell cancer.

## Introduction

It is estimated that there are 263,900 new cases and 128,000 deaths from oral cavity cancer in 2008 worldwide [1]. The increasing incidence and mortality rates of oral cancer in recent years pose a big challenge to doctors and scientists. Oral squamous cell carcinoma (OSCC) accounts for the majority of oral cancer. Both environmental factors and genetic factors play important roles in the development of oral cancer. It is well known that smoking is the predominant risk factor for oral cancer. A review highlighted the strength of the association between tobacco use and OSCC [2]. However, not all OSCC patients have a history of smoking and not all smoking persons develop OSCC, which suggests that the genetic susceptibility factors are likely involved in the etiology of OSCC. The identification of biomarkers for screening the high-risk individuals for increased predisposition to cancer is very important for prevention of OSCC.

Molecular epidemiologic studies showed that there were hundreds of genes involved in OSCC [3], in which there were some new genes. Although studying known genes could help to further understand the development of OSCC, newly developed markers such as noncoding small RNAs may lead novel insight into the molecular mechanisms that contribute to OSCC. More and more evidence supports a role for microRNAs (miRNAs) in cancer development, but there are few reports on OSCC. MiRNAs are a class of small non-coding RNAs of approximately 20 nucleotides in length, which are considered to regulate gene expression by binding to its 3’-UTR regions [4]. To date, emerging evidence has demonstrated that miRNAs play important roles in a broad range of physiologic and pathologic processes, such as differentiation, apoptosis, proliferation and development of various diseases including cancers [5–7]. Although the biologic functions of microRNAs remain largely unclear, there are evidence that more than half of miRNA genes located in cancer-related genomic regions or among fragile sites, suggesting that miRNAs may participate in the pathogenesis of human cancers [8]. Published results have indicated that microRNAs may be crucial factors in oncogenesis, functioning as tumor suppressors and/or oncogenes, and affect the etiology of various cancers [9–12]. Therefore, inherited genetic variations of microRNAs may affect the susceptibility to OSCC.

The most widely studied genetic alterations in recent years are single nucleotide polymorphisms (SNPs). SNPs within the seed region or in the precursor stem-loop of miRNA may significantly affect the production or processing of miRNA [13–14]. Now the researchers conclude that SNPs or mutations in sequences of miRNAs may affect their expression, maturation and/or target selection, and consequently influence cancer risks. In recent years, the association between polymorphisms in flanking regions of miRNAs and the risk of cancer has become the hot topic in cancer etiology research. However, the effect of the SNPs in miRNAs on the susceptibility of OSCC in the population of Chia has not been reported so far. In the present study, three SNPs in three miRNAs were analyzed. Through an extensive exploring of the databases of the International HapMap Project [15], dbSNP [16], and miRBase registry [17], as well as considering the minor allele frequency of selected SNPs in Asian population, we identified three potential functional polymorphisms in three pre-miRNAs (miR-196a2 rs11614913, miR-146a rs2910164 and miR-499 rs3746444). To our knowledge, this is the first case-control study of OSCC in a northeast Chinese population, to evaluate the roles of the SNPs in pre-miRNAs on risk of oral cancer.

## Materials and methods

### Study subjects

This is a hospital-based case-control study of oral cancer in Shenyang City, located in northeast China. The case group consisted of 340 pathologically confirmed diagnosed OSCC patients (between January 2012 and September 2014) at Hospital of Stomatology, China Medical University. During the same period, 340 controls were recruited from other patients with no evidence or history of cancer in the same institute. Controls were frequency matched to cases on age (±5 years) and same sex. Participants were unrelated ethnic Han Chinese. Each participant contributed 10ml venous blood and was interviewed to collect related data at the time they were admitted into the study. The written informed consent was obtained from each participant, and human investigations were approved by the Institutional Review Board of Hospital of Stomatology, China Medical University.

### SNP genotyping

Genomic DNA samples of all cases and controls were isolated by Phenol-chloroform Method. Genotyping of the miRNA SNPs was done using Taqman allelic discrimination method (Applied Biosystems, Foster City, CA) on an Applied Biosystems 7500 FAST Real-Time PCR System (Foster City, CA, USA). The commercially available primer probe sets (assay ID C_15946974_10 for rs2910164, C_31185852_10 for rs11614913, and C_2142612_30 for rs3746444) were adopted in PCR amplification. When genotyping was performed, appropriate negative controls were included in each run. Ten percent of participants were randomly selected as the masked duplicate sets to be genotyped twice by different persons, and the results were found to be concordant for all of them.

### Statistical analysis

The differences in distribution of demographic factors and tobacco smoking between cases and controls were examined using Pearson’s chi-square test and Student’s t-test. The goodness-of-fit χ^2^ tests were adopted to test the Hardy-Weinberg equilibrium (HWE) of the SNPs. was The odds ratios (OR) and their 95% confidence intervals (CI) to evaluate the associations between SNPs and oral cancer risks were calculated using unconditional logistic regression analyses. The association of miRNA polymorphisms and oral cancer risk stratified by sex were analyzed. The joint effects of miRNA polymorphisms and smoking on the risk of oral cancer were assessed by crossover analysis models. All of the tests were two-sided and statistical significance was defined as P<0.05. Multiple comparison tests (Bonferroni correction) were used and statistically significant P value threshold were defined as 0.017 after multiple comparison adjustment. All statistical analyses were performed using SPSS 20.0 software (SPSS, Inc. Chicago, IL, USA).

## Results

There are 340 cases and 340 controls included in the present study. The mean ages were 54.7±10.8 and 55.6±10.6 in case and control group and there was no difference in mean age between two groups (t = 1.009, P = 0.313). The chi-square tests showed the distribution of sex was equilibrium in cases and controls (X^2^ = 0.314, P = 0.575). The smokers were more likely to develop OSCC (OR = 2.075, 95%CI = 1.523–2.827, P<0.001). The distribution of demographic factors and tobacco smoking in cases and controls are shown in Table 1. The observed genotype frequencies for the three polymorphisms were all in agreement with that expected under the Hardy-Weinberg equilibrium in the controls (P = 0.530 for miR-146a rs2910164, P = 0.106 for miR-196a2 rs11614913, P = 0.633 for miR-499 rs3746444).

**Table 1:** Distribution of demographic factors and tobacco smoking in cases and controls.

Variable	Case (%)	Control (%)	P value
Age (years)	54.7±10.8	55.6±10.6	0.313
<41	43(12.6)	34(10.0)	
41–50	66(19.4)	69(20.3)	
51–60	110(32.4)	105(30.9)	
>60	121(35.6)	132(38.8)	0.634
sex			
Male	270(79.4)	264(77.6)	
Female	70(20.6)	76(22.4)	0.575
Tobacco smoking			
Ever	225(66.2)	165(48.5)	
Never	115(33.8)	175(51.5)	<0.001

Table 2 showed the association between the four SNPs and OSCC risks. For miR-499 rs3746444, individuals carrying homozygous CC genotype had increased risks of oral cancer compared with the homozygous wild TT genotype (adjusted OR was 3.154, 95%CI was 1.555–6.397, P value was 0.001). We found that the rs3746444 heterozygote TC and variant homozygote CC of miR-499 were associated with a higher risk of OSCC compared with its wild homozygote TT (adjusted OR = 1.441, 95%CI = 1.052–1.974, P = 0.023) but the result was not statistically significant after Bonferroni correction, showing the SNP appears to be dominant. The result also suggests the SNP is likely to be recessive and the individuals carrying CC genotype were more likely to develop OSCC than those with TT or TC genotype (adjusted OR = 2.903, 95%CI = 1.446–5.825, P = 0.003). Allele comparison showed that the C allele of miR-499 rs3746444 was associated with a higher risk of OSCC with significant odds ratio of 1.453 (95%CI = 1.127–1.874, P = 0.004). However, in analyses of miR-196a2 rs11614913 and miR-146a rs2910164, we have not found the significant associations with OSCC risks in any models.

**Table 2:** Allele and genotype frequencies of miRNA polymorphisms among cases and controls as well as their effects on oral cancer risk.

SNP	Oral cancer cases (%)	Controls (%)	OR (95%CI)*	P value
miR-196a2 rs11614913				
TT(Ref.)	100(29.4)	97(28.5)	-	
TC	169(49.7)	155(45.6)	1.010(0.704–1.451)	0.955
CC	71(20.9)	88(25.9)	0.735(0.479–1.129)	0.160
TC+CC vs TT	240(70.6)	243(71.5)	0.911(0.649–1.279)	0.590
CC vs TT+TC	269(79.1)	252(74.1)	0.731(0.508–1.051)	0.091
T allele(Ref.)	369(54.3)	349(51.3)	-	
C allele	311(45.7)	331(48.7)	0.889(0.718–1.100)	0.277
miR-146a rs2910164				
CC(Ref.)	189(55.6)	207(60.9)	-	
CG	124(36.5)	114(33.5)	1.217(0.876–1.690)	0.241
GG	27(7.9)	19(5.6)	1.738(0.923–3.271)	0.087
CG+GG vs CC	151(44.4)	133(39.1)	1.288(0.944–1.759)	0.111
GG vs CC+CG	313(92.1)	321(94.4)	1.614(0.868–3.000)	0.130
C allele(Ref.)	502(73.8)	528(77.6)	-	
G allele	178(26.2)	152(22.4)	1.232(0.961–1.579)	0.100
miR-499 rs3746444				
TT(Ref.)	191(56.2)	217(63.8)	-	
TC	118(34.7)	111(32.6)	1.258(0.904–1.752)	0.174
CC	31(9.1)	12(3.5)	3.154(1.555–6.397)	0.001
TC+CC vs TT	149(43.8)	123(36.2)	1.441(1.052–1.974)	0.023
CC vs TT+TC	309(90.9)	328(96.5)	2.903(1.446–5.825)	0.003
T allele(Ref.)	500(73.5)	545(80.1)	-	
C allele	180(26.5)	135(19.9)	1.453(1.127–1.874)	0.004

Abbreviation: SNP, single nucleotide polymorphism; OR, odds ratio; CI, confidence interval.

*ORs were calculated by unconditional logistic regression and adjusted for age, sex and smoking.

Further analyses were performed stratified by sex, and more significant results were found in males but not in females (Table 3). In males, comparing with rs2910164 homozygote genotype (CC) of miR-146a polymorphism, the CG genotype was associated with an increased OSCC risk (adjusted OR = 1.504, 95% CI = 1.038–2.180) and individuals with CG/GG genotype also had an increased susceptibility to OSCC (adjusted OR = 1.532, 95% CI = 1.078–2.178), but the results were not statistically significant after Bonferroni correction. Rs2910194 G allele of miR-146a appeared to be risk allele of OSCC in males (OR = 1.343, 95% CI = 1.015–1.778, P = 0.039) but the result was not statistically significant after Bonferroni correction. The similar results were found in the relationship between miR-499 rs3746444 polymorphism and OSCC risk in males. The results of CC vs TT, TC/CC vs TT, CC vs TT/TC or C allele vs T allele were significant and corresponding ORs were 2.989 (1.375–6.497), 1.550 (1.085–2.214), 2.682 (1.248–5.764) and 1.527 (1.143–2.041). But in females, we did not find any main effects between the three SNPs and OSCC risks (Table 3).

**Table 3:** The association of miRNA polymorphisms and oral cancer risk stratified by sex.

	SNP	Oral cancer cases(%)	Controls (%)	OR (95%CI)*	P value
Male	miR-196a2 rs11614913				
	TT(Ref.)	81(30.0)	82(31.1)	-	
	TC	134(49.6)	118(44.7)	1.086(0.727–1.621)	0.688
	CC	55(20.4)	64(24.2)	0.823(0.509–1.331)	0.428
	TC+CC vs TT	189(70.0)	182(68.9)	0.993(0.683–1.444)	0.971
	CC vs TT+TC	215(79.6)	200(75.8)	0.783(0.518–1.185)	0.247
	T allele(Ref.)	296(54.8)	282(53.4)	-	
	C allele	244(45.2)	246(46.6)	0.945(0.743–1.202)	0.645
	miR-146a rs2910164				
	CC(Ref.)	144(53.3)	165(62.5)	-	
	CG	105(38.9)	83(31.4)	1.504(1.038–2.180)	0.031
	GG	21(7.8)	16(6.1)	1.504(0.834–3.386)	0.147
	CG+GG vs CC	126(46.7)	99(37.5)	1.532(1.078–2.178)	0.017
	GG vs CC+CG	249(92.2)	248(93.9)	1.439(0.725–2.857)	0.298
	C allele(Ref.)	393(72.8)	413(78.2)	-	
	G allele	147(27.2)	115(21.8)	1.343(1.015–1.778)	0.039
	miR-499 rs3746444				
	TT(Ref.)	153(56.7)	174(65.9)	-	
	TC	92(34.1)	80(30.3)	1.370(0.940–1.997)	0.101
	CC	25(9.3)	10(3.8)	2.989(1.375–6.497)	0.006
	TC+CC vs TT	117(43.3)	90(34.1)	1.550(1.085–2.214)	0.016
	CC vs TT+TC	245(90.7)	254(96.2)	2.682(1.248–5.764)	0.011
	T allele(Ref.)	398(73.7)	428(81.1)	-	
	C allele	142(26.3)	100(18.9)	1.527(1.143–2.041)	0.004
Female	miR-196a2 rs11614913				
	TT(Ref.)	19(27.1)	15(19.7)	-	
	TC	35(50.0)	37(48.7)	0.639(0.266–1.534)	0.316
	CC	16(22.9)	24(31.6)	0.451(0.168–1.212)	0.114
	TC+CC vs TT	51(72.9)	61(80.3)	0.564(0.247–1.288)	0.174
	CC vs TT+TC	54(77.1)	52(68.4)	0.611(0.278–1.341)	0.219
	T allele(Ref.)	73(52.1)	67(44.1)	-	
	C allele	67(47.9)	85(55.9)	0.723(0.456–1.147)	0.168
	miR-146a rs2910164				
	CC(Ref.)	45(64.3)	42(55.3)	-	
	CG	19(27.1)	31(40.8)	0.552(0.260–1.174)	0.123
	GG	6(8.6)	3(3.9)	1.895(0.420–8.544)	0.405
	CG+GG vs CC	25(35.7)	34(44.7)	0.673(0.332–1.363)	0.271
	GG vs CC+CG	64(91.4)	73(96.1)	2.339(0.532–10.286)	0.261
	C allele(Ref.)	109(77.9)	115(75.7)	-	
	G allele	31(22.1)	37(24.3)	0.884(0.513–1.524)	0.657
	miR-499 rs3746444				
	TT(Ref.)	38(54.3)	43(56.6)	-	
	TC	26(37.1)	31(40.8)	1.001(0.488–2.053)	0.999
	CC	6(8.6)	2(2.6)	3.455(0.593–20.128)	0.168
	TC+CC vs TT	32(45.7)	33(43.4)	1.145(0.573–2.288)	0.702
	CC vs TT+TC	64(91.4)	74(97.4)	3.454(0.608–19.620)	0.162
	T allele(Ref.)	102(72.9)	117(77.0)	-	
	C allele	38(27.1)	35(23.0)	1.245(0.733–2.117)	0.417

Abbreviation: SNP, single nucleotide polymorphism; OR, odds ratio; CI, confidence interval.

*ORs were calculated by unconditional logistic regression and adjusted for age and smoking.

In addition, we examined the joint effects of miRNA polymorphisms and smoking on the risk of OSCC. As shown in Table 4, the association between microRNA genetic variants and OSCC risk was modified by smoking. Specifically, compared with individuals with both miR146a rs2910164 GG genotype and never smoking, those with both GC or CC genotype and never smoking had an increased risk of OSCC (OR = 1.811, 95% CI = 1.124–2.918), and the risk was even higher among those with both GC or CC genotype and ever smoking (OR = 2.713, 95%CI = 1.719–4.282). In contrast, compared with individuals with both miR-499 rs3746444 TT genotype and never smoking, those with both TC/CC genotype and never smoking had an increased risk of OSCC (OR = 2.247, 95% CI = 1.388–3.639), and the risk was even higher among those with both TC/CC genotype and ever smoking (OR = 3.013, 95%CI = 1.901–4.777). Moreover, in order to test whether the modification effect suggest an interaction, we further performed tests for interaction between smoking and microRNA variants for risk of OSCC. However, we found that the interaction between smoking and each of these microRNA variants on the risk of OSCC was not statistically significant (P values were 0.150, 0.088 and 0.050 for rs11614913, rs2910164 and rs3746444, respectively).

**Table 4:** The joint effects of miRNA polymorphisms and smoking on the risk of oral cancer.

Smoking	SNP	Cancer cases(%)	Controls (%)	OR (95%CI)*	P value
	miR-196a2 rs11614913				
Never	CC(Ref.)	47(13.8)	48(14.1)	-	
Never	CT+TT	68(20.0)	127(37.4)	0.556(0.337–0.917)	0.021
Smoking	CC	53(15.6)	49(14.4)	1.113(0.636–1.948)	0.708
Smoking	CT+TT	172(50.6)	116(34.1)	1.531(0.960–2.444)	0.074
	miR-146a rs2910164				
Never	GG(Ref.)	54(15.9)	108(31.8)	-	
Never	GC+CC	61(17.9)	67(19.7)	1.811(1.124–2.918)	0.015
Smoking	GG	135(39.7)	99(29.1)	2.719(1.791–4.128)	<0.001
Smoking	GC+CC	90(26.5)	65(19.4)	2.713(1.719–4.282)	<0.001
	miR-499 rs3746444				
Never	TT(Ref.)	53(15.6)	115(33.8)	-	
Never	TC+CC	62(18.2)	60(17.6)	2.247(1.388–3.639)	0.001
Smoking	TT	138(40.6)	102(30.0)	2.925(1.933–4.428)	<0.001
Smoking	TC+CC	87(25.6)	63(18.5)	3.013(1.901–4.777)	<0.001

Abbreviation: SNP, single nucleotide polymorphism; OR, odds ratio; CI, confidence interval.

*ORs were calculated by unconditional logistic regression and adjusted for age and sex.

## Discussion

In the present study, we evaluated the association between three functional polymorphisms in three common miRNAs and oral cancer risks in a Chinese population of 340 incident Asian OSCC cases and 340 healthy controls. To the best of our knowledge, this is the first study presenting the role of these three pre-miRNA polymorphisms together as well as their joint effect with tobacco smoking in the etiology of oral cancer in a northeastern Chinese population. Our findings suggest that the miR-499 rs3746444 polymorphism is associated with the risk of OSCC in Chinese, particularly among males.

MiRNAs may act as regulators of gene expression and be involved in many cellular processes. Therefore, functional genetic polymorphisms of miRNAs may lead to individual variations in many cellular processes, leading to modification of the risk of cancers. The studies have shown that some miRNAs are up-regulated, while other microRNAs are downregulated in oral cancer [18–19]. Such increased or decreased expression of microRNAs may be associated with the development, progression, and prognosis of oral cancer [20].

Recently, several studies have reported the relationships of miRNA SNPs and the risk of head and neck cancers. One study indicated that the miR-196a2 rs11614913 polymorphism was associated with the risk of head and neck cancers [21]. Liu et al. reported that miR-146a rs2910164 and miR-196a2 rs11614913 did not affect the risk of head and neck cancers but miR-499 rs3746444 moderately reduced the risk of head and neck cancers [22]. Another study showed that miRNA variants may modify the risk of HPV16-associated OSCC, particularly in never smokers [23]. The reasons for these conflicting results may be different tumor sites, small sample sizes, and lack of information such as other risk factors or confounders. For example, the previous studies, although with larger sample size than our current study, had mixed tumor sites and without stratification by sex, whereas in our present study, we only included the oral squamous cell cancer patients, performing the analyses stratified by male and female, as well as assessed the joint effects of miRNA SNPs and cigarette smoking on OSCC risk. Smoking has been identified as one of the primary etiologic factors for causes of OSCC. But in the present study, the number of female subjects is quite small and much smaller than the number of male subjects so it is possible that the insufficient numbers of female subjects were available for the study not that the SNP had no effect. Another reason may lead to the difference between the present result and previous published studies is the genotyping methods. The present study and the study performed by Christensen et al conducted Taqman allelic discrimination with the same commercially available primer probe set [21], however the other studies used PCR-restriction fragment length polymorphism method [22–23].

Because the frequency of the miRNA SNPs among the healthy controls is significantly different among different ethnicities, it is necessary to study the association of SNPs with cancer risk in diverse ethnic populations. So we underwent the present study in a Chinese population to reduce the risk of confounding due to population stratification and to provide the evidence of associations between miRNAs and oral cancer risks. Another major strength of the present study was the inclusion criteria for the controls. More and more evidence indicated that miRNAs were involved in some other diseases, which may be not clear. The control group in the current study included only healthy subjects, who were free of cancers or other chronic diseases, in order to exclude the effect of nonmalignant disease on the genotype distribution in the controls. In addition, because the age distribution of non-participating controls were similar to those of the participating ones, self-selection bias was unlikely. Moreover, the genotype distribution for each SNP was accordant with HWE, which further supports the randomness of our control subjects. Taken together, these strengths increased the reliability of our finding about an association between the miRNA polymorphisms and OSCC risks.

In this case-control study, several limitations need to be addressed. Firstly, the cases and controls were chosen from hospitals and may not fully represent the general population which may result in selection bias in the study. So we should be cautious when we reached a conclusion. Secondly, because we selected only oral squamous cell cancer patients as our study population and excluded other head and neck cancer patients in order to control some confounding bias, the sample size could not be very large and statistical power of our study was limited, it is just as well that that the control group is as large as the case group; therefore it is possible to reach a more statistical power. Finally, the current study lacked the biological and functional research to determine whether the genetic variants identified in our studies modulate oral cancer risk through their influences on the functions or expressions of their host genes.

In summary, this study firstly reported the association between single nucleotide polymorphisms in miRNAs and risks of oral squamous cell cancer in northeastern Chinese. Our study would have a certain positive significance in the understanding of oral cancer pathogenesis. Future larger studies with other ethnic populations and functional analysis are required to confirm current findings.

## Conclusion

The miR-146a rs2910164 and miR-499 rs3746444 polymorphisms might alter individual susceptibility to oral squamous cell cancer in Chinese.
